# Association between Serum Tissue Inhibitor of Matrix Metalloproteinase-1 Levels and Mortality in Patients with Severe Brain Trauma Injury

**DOI:** 10.1371/journal.pone.0094370

**Published:** 2014-04-11

**Authors:** Leonardo Lorente, María M. Martín, Patricia López, Luis Ramos, José Blanquer, Juan J. Cáceres, Jordi Solé-Violán, Jorge Solera, Judith Cabrera, Mónica Argueso, Raquel Ortiz, María L. Mora, Santiago Lubillo, Alejandro Jiménez, Juan M. Borreguero-León, Agustín González, Josune Orbe, José A. Rodríguez, José A. Páramo

**Affiliations:** 1 Intensive Care Unit, Hospital Universitario de Canarias, Santa Cruz de Tenerife, Spain; 2 Intensive Care Unit, Hospital Universitario Nuestra Señora de Candelaria, Santa Cruz de Tenerife, Spain; 3 Intensive Care Unit, Hospital General La Palma, La Palma, Spain; 4 Intensive Care Unit, Hospital Clínico Universitario de Valencia, Fundación INCLIVA, Valencia, Spain; 5 Intensive Care Unit, Hospital Insular, Las Palmas de Gran Canaria, Spain; 6 Intensive Care Unit, Hospital Universitario Dr. Negrín, Las Palmas de Gran Canaria, Spain; 7 Deparment of Anesthesiology and Reanimation, Hospital Universitario de Canarias, Santa Cruz de Tenerife, Spain; 8 Research Unit, Hospital Universitario de Canarias, Santa Cruz de Tenerife, Spain; 9 Laboratory Department, Hospital Universitario de Canarias, Santa Cruz de Tenerife, Spain; 10 Atherosclerosis Research Laboratory, CIMA-University of Navarra, Pamplona, Spain; University of Patras, Greece

## Abstract

**Objective:**

Matrix metalloproteinases (MMPs) and tissue inhibitors of matrix metalloproteinases (TIMPs) play a role in neuroinflammation after brain trauma injury (TBI). Previous studies with small sample size have reported higher circulating MMP-2 and MMP-9 levels in patients with TBI, but no association between those levels and mortality. Thus, the aim of this study was to determine whether serum TIMP-1 and MMP-9 levels are associated with mortality in patients with severe TBI.

**Methods:**

This was a multicenter, observational and prospective study carried out in six Spanish Intensive Care Units. Patients with severe TBI defined as Glasgow Coma Scale (GCS) lower than 9 were included, while those with Injury Severity Score (ISS) in non-cranial aspects higher than 9 were excluded. Serum levels of TIMP-1, MMP-9 and tumor necrosis factor (TNF)-alpha, and plasma levels of tissue factor (TF) and plasminogen activator inhibitor (PAI)-1 plasma were measured in 100 patients with severe TBI at admission. Endpoint was 30-day mortality.

**Results:**

Non-surviving TBI patients (n = 27) showed higher serum TIMP-1 levels than survivor ones (n = 73). We did not find differences in MMP-9 serum levels. Logistic regression analysis showed that serum TIMP-1 levels were associated 30-day mortality (OR = 1.01; 95% CI = 1.001–1.013; P = 0.03). Survival analysis showed that patients with serum TIMP-1 higher than 220 ng/mL presented increased 30-day mortality than patients with lower levels (Chi-square = 5.50; *P* = 0.02). The area under the curve (AUC) for TIMP-1 as predictor of 30-day mortality was 0.73 (95% CI = 0.624–0.844; P<0.001). An association between TIMP-1 levels and APACHE-II score, TNF- alpha and TF was found.

**Conclusions:**

The most relevant and new findings of our study, the largest series reporting data on TIMP-1 and MMP-9 levels in patients with severe TBI, were that serum TIMP-1 levels were associated with TBI mortality and could be used as a prognostic biomarker of mortality in TBI patients.

## Introduction

Traumatic brain injury (TBI) is an important cause of disability, mortality and cost [1]. Primary injury refers to the initial physical forces applied to brain during the impact. Secondary injury occurs over a period of hours or days following the initial traumatic injury, and is developed by different mediators. During the secondary injury there is an increase in the permeability of blood-brain-barrier (BBB) and consequently appears brain edema [2]–[5].

Matrix metalloproteinases (MMPs) are a family of zinc-containing endoproteinases implicated in degradation and remodelling of the extracellular matrix (ECM). They can be classified broadly by substrate specificity into: collagenases (MMP-1, -8 and -13), gelatinases (MMP-2 and -9), stromelysins (MMP-3, -10, -11), elastases (MMP-7 and -12) and membranetype (MT-MMPs, MMP-14, -15, -16 and -17). The regulation of its activity is complex and occurs at several levels [6], such as transcriptional (MMP gene expression in cells) that is influenced by numerous stimulatory and suppressive factors that influence multiple signalling pathways, as TNF-α, interleukin-1β, transforming growth factor (TGF)-β, TGF-α; posttranscriptional (stability of MMP transcripts in cells) that is influenced by glucocorticoids and TGF-β; translational (release of MMP from cells) that is influenced by plasmin and thrombin; and post-translational (activation of MMPs after the release) that is influenced by oxidative stress, nitrosidative stress, phosphorylation, proteolysis and tissue inhibitors of matrix metalloproteinases (TIMPs). We and others have shown that MMPs have a role in normal physiological functions such as the menstrual cycle, morphogenesis, tissue remodelling and angiogenesis, and in diseases with abnormal ECM turnover, such as arthritis, sepsis, tumour invasion, aneurysm formation and atherosclerosis [7]–[11]. In addition, MMPs play a role in central nervous system and are involved in the mechanisms associated with neuroinflammation [12]–[14]. Several studies have inmunolocalized MMP in the human brain, specifically in astrocytes, microglia and neurons [14]. The findings of several animal studies suggested that MMP are involved in the disruption of the BBB, edema formation and inflammation after central nervous system trauma [15]–[17].

Previous studies with small sample size (fewer than 50 patients) have reported higher circulating levels of MMP-2 and MMP-9 in patients with TBI than in healthy control subjects [18]–[24]. Also there has been found higher levels of MMP-2 and MMP-9 in brain extracellular fluid (ECF), indicating that both local and systemic trauma-induced upregulation of MMPs play an important role in the pathophysiology of TBI [15], [18]. However, an association between MMP levels, severity and mortality of patients with TBI was not reported. In a previous study by our team, we found higher serum TIMP-1 and lower serum MMP-9 levels in non-surviving than in surviving severe septic patients [9]. Thus, the objective of this study was to determine whether serum levels of TIMP-1 and MMP-9 are associated with severity and mortality of patients with severe TBI.

## Methods

### Design and Subjects

This is a multicenter, observational, prospective study carried out in 6 Intensive Care Units of Spain. The study was approved by the Institutional Review Board of the 6 participant hospitals: Hospital Universitario de Canarias (La Laguna, Santa Cruz de Tenerife, Spain), Hospital Universitario Nuestra Señora de Candelaria (Santa Cruz de Tenerife, Spain), Hospital General de La Palma (La Palma, Spain), Hospital Clínico Universitario de Valencia (Valencia, Spain), Hospital Insular (Las Palmas de Gran Canaria, Spain), Hospital Universitario Dr. Negrín (Las Palmas de Gran Canaria, Spain). The written informed consent from the patients or from their legal guardians was obtained.

We included 100 patients with severe TBI. Severity of brain trauma injury was classified according to Glasgow Coma Scale (GCS) [25], and severe TBI was defined as GCS lower than 9 points.

Exclusion criteria were: age less than 18 years, pregnancy, inflammatory or malignant disease and Injury Severity Score (ISS) [26] in non-cranial aspects higher than 9 points.

### Variables recorded

The following variables were recorded for each patient: sex, age, ISS, GCS, lactic acid, platelets, international normalized ratio (INR), activated partial thromboplastin time (aPTT), fibrinogen, Acute Physiology and Chronic Health Evaluation II (APACHE II) score [27] and brain lesion according to the Marshall computer tomography (CT) classification [28].

### End-point

The end-point was 30-day mortality.

### Blood sample collection

Blood samples in 100 patients with severe TBI were collected in the day of TBI to measure serum levels of TIMP-1 and MMP-9. We also measured tumor necrosis factor (TNF)-alpha serum levels to assess inflammation, and plasma levels of tissue factor (TF) to assess coagulation and plasminogen activator inhibitor (PAI)-1 as a fibrinolytic marker.

### Determination of TIMP-1, MMP-9 and TNF-alpha serum levels

Serum separator tubes were used to determine serum TIMP-1 and MMP-9 levels. Venous blood samples were taken and centrifuged within 30 minutes at 1000 g for 15 min, and the serum was removed and frozen at −80°C until measurement.

TIMP-1 and MMP-9 assays were performed at the Atherosclerosis Research Laboratory of CIMA-University of Navarra (Pamplona, Spain) and were assayed by specific ELISAs (Quantikine, R&D Systems, Abingdon, United Kingdom) according to the manufacturer's instructions with a serum dilution of 1∶100 and 1∶80 respectively. This test has been validated by other systems in serum samples [29]. The interassay coefficients of variation (CV) were <8% (n = 20) and detection limit for the assays were 0.15 ng/mL and 0.31 ng/mL respectively.

TNF-alpha serum levels were measured in the Laboratory Deparment of the Hospital Universitario de Canarias (La Laguna, Santa Cruz de Tenerife, Spain) by a solid-phase, chemiluminiscents immunometrics assays kit (Immulite, Siemens Healthcare Diagnostics Products, Llanberis, United Kingdom); and the interassays coefficient of variation (CV) was <6.5% (n = 20) and detection limit for the assay was 1.7 pg/mL.

### Determination of TF and PAI-1 plasma levels

Venous blood samples were collected in citrate collected plasma tubes and centrifugedwithin 30 minutes at 1000*g for 15 min. The plasma was removed and frozen at −80°C until measurement. TF and PAI-1 assays were performed at the Laboratory Department of the Hospital Universitario de Canarias (La Laguna, Santa Cruz de Tenerife, Spain). TF levels were assayed by specific ELISA (Imubind Tissue Factor ELISATM, American Diagnostica, Inc, Stanford, CT, USA). PAI-1 antigen levels were assayed by specific ELISA (Imubind Plasma PAI-1 ElisaTM, American Diagnostica, Inc, Stanford, CT, USA). The interassay coefficients of variation (CV) of TF and PAI-1 assays were <8% (n = 20) and <5% (n = 20) respectively, and detection limits for the assays were 10 pg/mL and 1 ng/mL respectively.

### Statistical Methods

Continuous variables are reported as medians and interquartile ranges. Categorical variables are reported as frequencies and percentages. Comparisons of continuous variables between groups were carried out using Wilcoxon-Mann-Whitney test. Comparisons between groups on categorical variables were carried out with chi-square test. Multiple binomial logistic regression analysis was applied to prediction of 30-day mortality. As number of events was 27 exitus, we constructed two multiple binomial logistic regression models with only three predictor variables in each to avoid an over fitting effect that may lead to choose a final model of order slightly higher order than required [30]. In the first model were included serum TIMP-1 levels, APACHE-II score and CT classification. Previously to include the variable CT classification in the regression analysis, it was recoded according with the risk of death observed in the bivariated analysis as low (CT types 2 and 5) and high risk (CT types 3, 4 and 6) of death. In the second model were included serum TIMP-1 levels, GCS and age. Odds Ratio and 95% confidence intervals were calculated as measurement of the clinical impact of the predictor variables. Receiver operating characteristic (ROC) analysis was carried out to determine the goodness-of-fit of the of serum TIMP-1 levels to predict 30-day mortality. Kaplan-Meier analysis of survival at 30 days and comparisons by log-rank test were carried out using serum TIMP-1 levels lower/higher than 220 ng/mL as the independent variable and survival at 30 days as the dependent variable. The association between continuous variables was carried out using Spearmańs rank correlation coefficient, and Bonferroni correction was applied to control for the multiple testing problem. A *P* value of less than 0.05 was considered statistically significant. Statistical analyses were performed with SPSS 17.0 (SPSS Inc., Chicago, IL, USA) and NCSS 2000 (Kaysville, Utah) and LogXact 4.1, (Cytel Co., Cambridge, MA).

## Results

Non-surviving TBI patients (n = 27) showed lower GCS, higher age and female rate, and APACHE-II score than survivors (n = 73). We found statistically significant differences in CT classification between non-surviving and surviving patients. In addition, non-surviving patients showed higher TIMP-1 levels than surviving. There were not significant differences between non-surviving and surviving patients in circulating levels of MMP-9 and TNF-alpha, TF and PAI-1 (Table 1).

**Table 1:** Baseline clinical and biochemical characteristics of survivor and non-survivor patients.

	Survivors (n = 73)	Non-survivors (n = 27)	P value
Gender female – n (%)	12 (16.4)	11 (40.7)	0.02
Age (years) - median (p 25-75)	47 (32–67)	66 (45–76)	<0.001
Computer tomography classification - n (%)			0.002
Type 1	0	0	
Type 2	21 (28.8)	3 (11.1)	
Type 3	13 (17.8)	5 (18.5)	
Type 4	10 (13.7)	6 (22.2)	
Type 5	26 (35.6)	5 (18.5)	
Type 6	3 (4.1)	8 (29.6)	
Temperature (°C) - median (p 25–75)	37. (35.6–37.3)	36.0 (35.0–37.0)	0.12
Sodium (mEq/L)- median (p 25–75)	139 (138–142)	141 (135–149)	0.19
Glycemia (g/dL) - median (p 25–75)	139 (120–163)	161 (142–189)	0.08
Leukocytes - median*10^3^/mm^3^ (p 25–75)	14.7 (10.2–19.3)	18.3 (10.7–23.9)	0.46
PaO2 (mmHg) - median (p 25–75)	151 (116–217)	141 (104–186)	0.34
PaO2/FI0_2_ ratio - median (p 25–75)	336 (242–407)	190 (154–316)	0.11
Bilirubin (mg/dl) - median (p 25–75)	0.50 (0.40–0.87)	0.75 (0.53–1.05)	0.045
Creatinine (mg/dl) - median (p 25–75)	0.80 (0.70–0.90)	0.95 (0.70–1.10)	0.44
Hemoglobin (g/dL) - median (p 25–75)	11.4 (10.4–13.0)	11.1 (9.4–12.3)	0.87
Glasgow Coma Scale score - median (p 25–75)	7 (6–8)	3 (3–6)	<0.00171
Lactic acid (mmol/L) median (p 25–75)	1.70 (1.23–2.50)	1.90 (1.15–4.55)	0.16
Platelets - median*10^3^/mm^3^ (p 25–75)	182 (143–252)	215 (139–264)	0.48
INR - median (p 25–75)	1.03 (0.92–1.15)	1.22 (1.01–1.67)	0.15
aPTT (seconds) - median (p 25–75)	28 (25–32)	26 (25–31)	0.86
Fibrinogen (mg/dl) - median (p 25–75)	350 (282–444)	376 (246–560)	0.32
APACHE-II score - median (p 25–75)	19 (17–23)	26 (25–32)	<0.001
ISS - median (ppe 25–75)	25 (25–32)	25 (25–27)	0.24
ICP (mmHg) - median (p 25–75)	15 (14–20)	20 (12–30)	0.27
CPP (mmHg) - median (p 25–75)	68 (57–70)	60 (54–69)	0.46
TIMP-1 (ng/mL) - median (p 25–75)	219 (177–258)	302 (221–474)	<0.001
MMP-9 (ng/mL) - median (p 25–75)	760 (428–1113)	948 (357–1180)	0.62
TNF-alpha (pg/mL) - median (p 25–75)	9.72 (7.88–13.40)	13.65 (8.35–22.75)	0.12
Tissue Factor (pg/mL) - median (p 25–75)	189 (129–247)	194 (169–295)	0.18
PAI-1 (ng/mL) - median (p 25–75)	64 (32–89)	65 (39–99)	0.99

P 25–75 = percentile 25^th^–75^th^; PaO_2_ = pressure of arterial oxygen/fraction inspired oxygen; FIO_2_  =  pressure of arterial oxygen/fraction inspired oxygen; ISS  =  Injury Severity Score; INR  =  international normalized ratio; aPTT  =  activated partial thromboplastin time; APACHE II  =  Acute Physiology and Chronic Health Evaluation; ICP  =  intracranial pressure; CPP  =  cerebral perfusion pressure; TIMP  =  tissue inhibitor of matrix metalloproteinase; MMP  =  matrix metalloproteinase; TNF  =  tumor necrosis factor; PAI  =  plasminogen activator inhibitor

Multiple binomial logistic regression analysis showed that serum TIMP-1 could predict 30-day mortality (OR = 1.01; 95% CI = 1.001–1.013; P = 0.03) controlling for APACHE-II and CT classification (Table 2). We found a mortality rate of 3/24 (12.5%) in patients with CT classification type 2, 5/18 (27.8%) with type 3, 6/16 (37.5%) with type 4, 5/31 (16.1%) with type 5 and 8/11 (72.7%) with type 6. Previously to include the variable CT classification in the regression analysis, it was recoded according with the risk of death observed in the bivariated analysis as low and high risk of death. As low risk of death were included patients with CT classification types 2 and 5, with a mortality rate of 8/55 (14.5%). As high risk of death were included patients with CT classification types 3, 4 and 6, with a mortality rate of 19/45 (42.2%).

**Table 2:** Multiple binomial logistic regression analysis of variables to predict 30-day mortality.

Variable	Odds Ratio	95% Confidence Interval	*P*
**First Model**			
TIMP-1 levels	1.01	1.001–1.0127	0.03
APACHE-II score	1.31	1.147–1.499	<0.001
Computer tomography classification (reference category: low risk of death)	7.65	1.976–29.616	0.003
**Second Model**			
TIMP-1 levels	1.01	1.003–1.015	0.002
GCS score	0.57	0.403–0.798	0.001
Age	1.10	1.044–1.149	<0.001

TIMP  =  tissue inhibitor of matrix metalloproteinase; APACHE II  =  Acute Physiology and Chronic Health Evaluation; GCS Glasgow Coma Scale

Multiple binomial logistic regression analysis also showed that serum TIMP-1 could predict 30- day mortality (OR = 1.01; 95% CI = 1.003–1.015; P = 0.002) controlling for GCS and age (Table 2).

**Figure 1:**
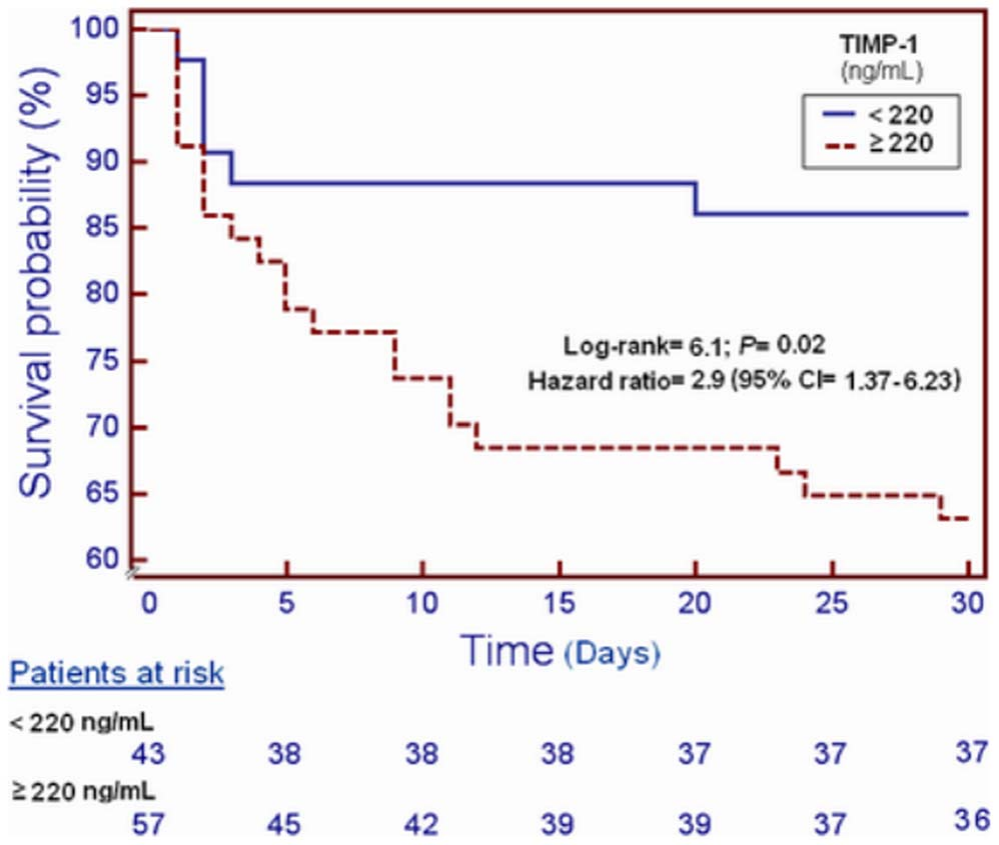
Survival curves at 30 days using 220/mL of TIMP-1 serum levels as cut-off.

Survival analysis showed that patients with serum TIMP-1 higher than 220 ng/mL presented higher 30-day mortality than patients with lower levels (Chi-square: 5.50; *P* = 0.02) (Figure 1).

**Figure 2:**
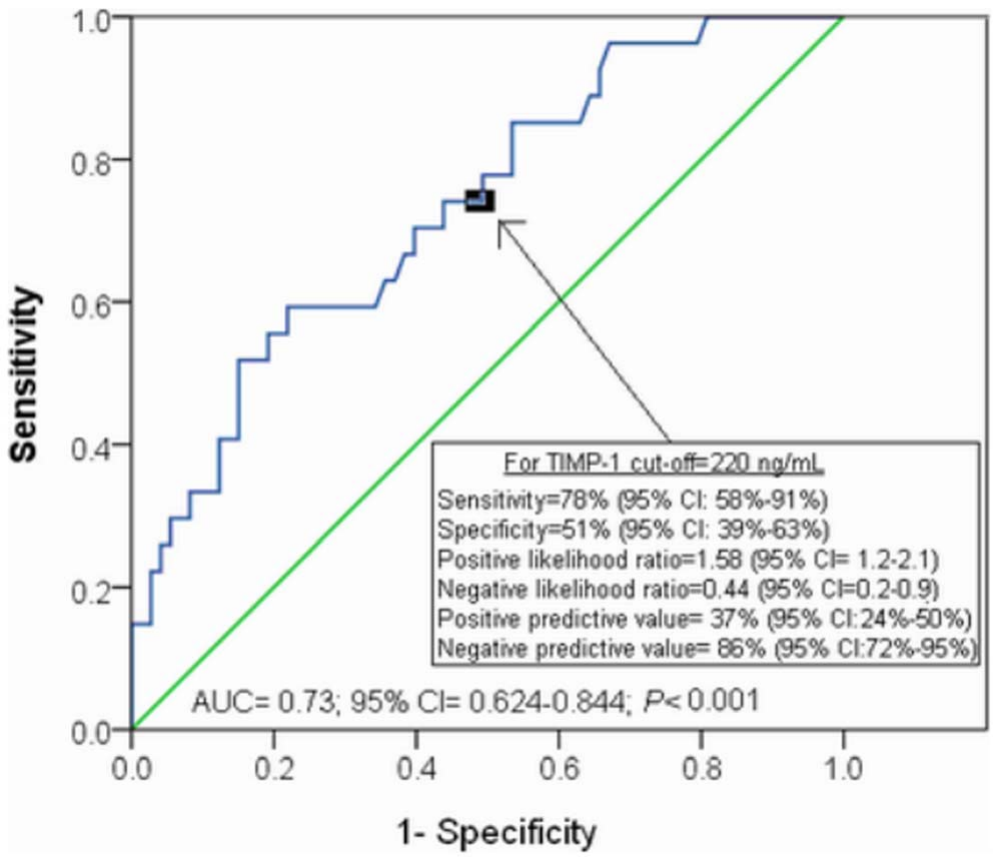
Receiver operation characteristic analysis using TIMP-1 serum levels as predictor of mortality at 30 days.

The area under the curve (AUC) for TIMP-1 as predictor of 30-day mortality was 0.73 (95% CI = 0.624–0.844; P<0.001) (Figure 2).

We found an association between TIMP-1 and APACHE-II (rho = 0.33; P = 0.001), TF (rho = 0.43; P<0.001) and TNF-alpha (rho = 0.43; P<0.001) (Table 3).

**Table 3:** Correlation between serum TIMP-1 levels and other baseline clinical and biochemical characteristics.

	TIMP-1 (ng/mL)
APACHE-II score	rho = 0.33; P = 0.001
GCS score	rho = −0.22; P = 0.03
Lactic acid (mmol/L)	rho = 0.24; P = 0.02
TNF-alpha (pg/mL)	rho = 0.53; P<0.001
Tissue Factor (pg/mL)	rho = 0.43; P<0.001
PAI-1 (ng/mL)	rho = 0.06; P = 0.71
Platelets (cells/mm^3^)	rho = −0.05; P = 0.61
INR	rho = 0.10; P = 0.35
aPTT (seconds)	rho = 0.08; P = 0.49
Fibrinogen (mg/dL)	rho = 0.07; P = 0.59
MMP-9 (ng/mL)	rho = 0.02; P = 0.88

TIMP =  tissue inhibitor of matrix metalloproteinase; GCS =  Glasgow Coma Scale; TNF  =  tumor necrosis factor; PAI  =  plasminogen activator inhibitor; INR  =  international normalized ratio; aPTT  =  activated partial thromboplastin time; MMP  =  matrix metalloproteinase. Bonferroni correction to control the multiple testing problem (0.05/11 = 0.004) was used. Only *P*-values lower than 0.004 were considered statistically significant.

## Discussion

To our knowledge, this study includes the largest series reporting data on MMP levels in patients with severe TBI. The most relevant and new findings of our study were the following: a) non-surviving TBI patients had higher serum TIMP-1 levels than surviving ones, b) there was an association between serum TIMP-1 levels and TBI severity and mortality, c) serum TIMP-1 levels could be used as a prognostic biomarker of mortality in TBI patients.

Previous studies with small sample size (fewer than 50 patients) have reported higher circulating levels of MMP-2 and MMP-9 in patients with TBI than in healthy control subjects [18]–[24]. However, they did not report differences in circulating MMP levels between nonsurviving and surviving patients. We show, for the first time, that non-surviving TBI patients showed higher serum TIMP-1 levels than survivors at day 1; however, we did not find significant differences in MMP-9 serum levels. Another new finding of our study was the association between serum TIMP-1 levels and mortality in logistic regression analysis. Also, as previously reported, we found that APACHE-II [31], [32] and CT classification [33] were associated with mortality. Interestingly, we found for the first time that TIMP-1 levels could be used as biomarker of mortality according to the ROC analysis. Another new finding of our study was the association between TIMP-1 levels and TBI severity assessed by APACHE-II score.

The physiological role of higher TIMP-1 levels in non-survivors compared to survivors TBI patients is still unknown. It is possible that the increase in TIMP-1 levels in non-survivors may be a consequence of increased MMP-2 and MMP-9 levels in non-survivors during the initial phase of TBI in order to maintain the balance between MMPs and TIMPs activity. However, we have not determined MMP-2 levels to test this possible explanation and we only found a trend, not statistically significant, of higher MMP-9 levels in non-surviving than in surviving TBI patients. In one study by Jaworski with TBI rats was found an increase of TIMP-1 mRNA expression in astrocytes and of TIMP-2 mRNA expression in microglia and neurons, while the expression of TIMP-3 and TIMP-4 was unaltered [34]. In one study by Tejima et al in TBI mice was found lower MMP-9 levels and lower brain lesion volumes in mice overexpressing TIMP-1 compared to wild-type mice [35]. In some rodent models of brain injury, the treatment with MMP-1 inhibitor has attenuated MMP-2 and MMP-9 activity, barrier disruption and edema formation [15]–[17].

MMPs play a role in the neuroinflammation [12]–[14]. During the secondary injury there is an increase in the permeability of BBB and consequently appears brain edema [2]–[5]. The findings of several animal studies suggested that MMP are involved in the disruption of the BBB and edema formation after central nervous system trauma [15]–[17]. Interestingly, there has been reported that circulating TIMP-1 levels are associated with brain edema in ischemic stroke patients [36].

There has been described that coagulopathy after TBI is frequent and represents a powerful predictor related to prognosis [37]–[39]. This coagulopathy could present as hypo- and hypercoagulable state, and result in a variable degree of secondary injury via subsequent ischemic and hemorrhagic lesioning. The proposed underlying mechanisms may comprise the release of TF and activation of hyperfibrinolysis. Interestingly, we found for the first time an association between circulating TIMP-1 and TF levels. However, we did not find significant differences between surviving and non-surviving patients in coagulation data as platelet count, INR, aPTT, fibrinogen and PAI-1. The association between circulating TIMP-1 and TF levels could contribute in a procoagulant state and thus, an increase of secondary injury via subsequent ischemic lesioning.

TBI may result in a systemic inflammatory response syndrome (SIRS) due to the synthesis and leaking to the circulation of pro-inflammatory cytokines [40], [41]. This SRIS may cause capillary thrombosis, a single or multiple organ failure and, finally, contribute to mortality. Interestingly, we found an association between TIMP-1 and TNF-alpha levels; however, we did not find differences between surviving and non-surviving patients in TNFalpha levels. As previously mentioned, we also found an association between TIMP-1 serum levels and TF plama levels, and this may contribute to capillary thrombosis.

Taken together, these data suggest that TIMP-1 levels could play a rol in pathophysiology of TBI. It is possible that increased serum TIMP-1 levels in non-survivors TBI patients is not the cause of death in TBI patients, but only a biomarker associated with mortality.

From a therapeutic perspective, the development of modulators of MMP activity could be used as a new class of drugs for the treatment of patients with severe TBI for neuroprotection [15]–[17].

The strengths of our study are that it was a multicenter study (which increases the external applicability of results to other similar units) and the larger sample size in relation to previous studies (that allowed us to increase the accuracy of the analysed parameters and report for the first time an association between serum TIMP-1 levels and mortality). However, some limitations of our study should be recognized. First, we did not perform the analysis of other MMPs and TIMPs; and this could be interesting to describe serum levels of other MMPs and TIMPs, and ratios between them. Second, the measure of other inflammatory cytokines would be desirable in order to better evaluate the relationship between MMPs and inflammatory response in this set of patients. Third, we did not report data about the evolution of TIMPs and MMPs on the time to describe the evolution in non-surviving and surviving TBI patients. Fourth, we have not determined MMPs and TIMPs in cerebrospinal fluid (CSF) to analyze whether there are an association between serum and CSF levels. This could be interesting due to in a study by Grossetete et al were found higher MMP-9 levels in CSF (but not in plasma), higher MMP-2 in plasma (but not in CSF), and higher MMP-3 latent form in CSF (but not active form and neither in plasma) in TBI patients compared to control subjects [22]. Fifth, the determination of MMPs was carried out by ELISA, and could be interesting to determine its active form by gelatine zymography [42]. Sixth, the specificity of serum TIMP-1 levels to predict mortality is low, and this restricts the application as biomarker to discard survivor patients. Thus, additional studies are needed to confirm the finding of our study.

## Conclusions

The most relevant and new findings of our study, the largest series reporting data on TIMP-1 and MMP-9 levels in patients with severe TBI, were that serum TIMP-1 levels were associated with TBI mortality and could be used as a prognostic biomarker of mortality in TBI patients.
